# Cannabidiol (CBD) and Insomnia : Literature review

**DOI:** 10.1192/j.eurpsy.2022.2092

**Published:** 2022-09-01

**Authors:** T. Gutierrez Higueras, F. Calera Cortés, E.D. Servin López, L. Montes Arjona, S. Sainz De La Cuesta Alonso, S. Vicent Forés

**Affiliations:** 1 Reina Sofia University Hospital, Psychiatry, Córdoba, Spain; 2 Hospital Reina Sofía Córdoba, Psychiatry, Cordoba, Spain; 3 Hospital Reina Sofía Córdoba, Psychiatry, Córdoba, Spain

**Keywords:** Treatment, CBD, Insomnia, sleep

## Abstract

**Introduction:**

Cannabidiol (CBD) is one of 113 cannabinoids identified in cannabis plants. Considered as a psycho-inactive component, recently, the Court of Justice of the European Union published a ruling in which it establishes that cannabidiol extracted from the cannabis plant should not be considered a drug under the United Nations Single Convention on Narcotic Drugs of 1961. Due to increased publicity on social media of the supposed benefits of this product, in addition to the lack of clear regulations, it is becoming a widely used treatment for sleep disorders.

**Objectives:**

To analyse literature for the effect of CBD in sleep disturbances, emphasizing advantages and disadvantages of its use.

**Methods:**

We carried out a literature review in Pubmed choosing those articles focused on effect of CBD in sleep disturbances.

**Results:**

The review of the effect of CBD on sleep cycle suggest that medium to high doses increased REM sleep latency, and medium-low doses decreased REM sleep latency. No evidence of withdrawal syndrome was found with abrupt discontinuation of short-term treatment with CBD.

**Conclusions:**

Most of the literature revised shows that the data was taken by self-questionares to CBD users. Studies suggest that a short use of medium to hight doses of CBD may improve insomnia, however, combined use with THC may result in a decrease in slow wave sleep. Longitudinal research should be done in order to understand the clinical impact of CBD on sleep.

**Disclosure:**

No significant relationships.

